# A thank you note to our reviewers

**DOI:** 10.1002/jgf2.653

**Published:** 2023-11-14

We would like to express our deepest gratitude to all the individuals who have provided their valuable time and expertise to support Journal of General and Family Medicine. The Editorial Board wishes to acknowledge with gratitude the following Reviewers for reviewing manuscripts during the past year.

Abe, Masanori

Affoo, Rebecca

Akashi, Yusaku

Akimoto, Takashi

Akiyama, Yutaro

Aoki, Takuya

Asano, Midori

Azuma, Teruhisa

Barger, Laura K.

Bianco, Gabriele

Chen, Yen‐Yuan

Cheney, Kate

Draman, Nani

Feng, Jie

Fujiwara, Motoshi

Fukui, Sho

Fukumori, Norio

Funakoshi, Hiraku

Hamano, Jun

Harada, Taku

Harada, Yukinori

Haruta, Junji

Hase, Ryota

Haseda, Maho

Hashizume, Naoki

Hayashi, Tetsuro

Hinata, Yuki

Hirai, Yuji

Hirakawa, Yoshihisa

Hirayama, Yoko

Hirose, Kazuhito

Iinuma, Yoshitsugu

Ikeda, Kotaro

Imafuku, Rintaro

Imamura, Takeaki

Inagaki, Masatoshi

Isenberg‐Grzeda, Elie

Ishikawa, Shizukiyo

Ishikawa, Eiichi

Ishisaka, Mariko

Isono, Hiroki

Iwamuro, Masaya

Iwata, Hiroyoshi

Jindai, Kazuaki

Jones, C. Allyson

Kaibara, Toshiki

Kameoka, Junichi

Kanakubo, Yusuke

Kaneko, Makoto

Kanke, Satoshi

Kanno, Tetsuya

Kanzaki, Go

Kanzawa, Yohei

Kasugai, Daisuke

Kataoka, Hitomi

Kataoka, Yuki

Katayama, Kohta

Katayama, Yusuke

Kato, Koki

Katsuki, Naoko

Kawahara, Hiroaki

Kawakami, Yoshio

Kenzaka, Tsuneaki

Kimura, Takuma

Kise, Morito

Knudtzen, Fredrikke C.

Kobayashi, Hiroyuki

Kodama, Toyohiko

Kosaka, Shintaro

Kudoh, Rie

Kuroda, Kaku

Kuroda, Moe

Kutsuna, Satoshi

Leclercq, Alexandre

Li, Yu

Matsumoto, Tomohiro

Matsumura, Shinji

Matsuno, Kei

Matsuyama, Yasushi

Midlöv, Patrik

Mitsuhashi, Toshiharu

Miyagami, Taiju

Miyamori, Daisuke

Miyazaki, Kei

Miyoshi, Toru

Morales, Mignodel M.

Mori, Hirotake

Motohashi, Iori

Motomura, Kazuhisa

Murai, Kenji

Nagano, Ayano

Nagasaki, Kazuya

Naitou, Yoshiki

Nakagama, Yu

Nakayama, Kuniko

Narita, Masashi

Narumoto, Keiichiro

Naskar, Sagar

Nishimori, Hisakazu

Nishioka, Daisuke

Nishisako, Hisashi

Nishizaki, Yuji

Obika, Mikako

Ogawa, Taku

Ohta, Ryuichi

Okada, Tadao

Okamoto, Mitsuhiro

Okazaki, Yuji

Omae, Kenji

Onishi, Hirotaka

Oshimi, Takayuki

Ota, Atsuhiko

Ozone, Sachiko

Ryuge, Akihiro

Sada, Ken‐ei

Sakaguchi, Yusuke

Sakai, Kana

Sakakura, Kenichi

Saloheimo, Pertti

Saraya, Takeshi

Sasaki, Yosuke

Sato, Shuntaro

Satoi, Yoshinao

Sechi, Gian Pietro

Sethi, Kalyan

Shehata, Sameh

Shibata, Ayako

Shikino, Kiyoshi

Shimizu, Ikuo

Shukla, Samarth

Son, Daisuke

Sugihara, Yuusaku

Sugimoto, Ryu

Suyama, Yasuhiro

Suzuki, Tomoharu

Takahashi, Noriyuki

Takahashi, Yuji

Takeda, Yoheii

Takeshita, kenichi

Tamiya, Nanako

Tanaka, Kendo

Tokuda, Koichi

Tokumasu, Kazuki

Torigoe, Kazuhiro

Tracy, Marguerite

Tsuchida, Tomoya

Tsutsumi, Akizumi

Ukawa, Shigekazu

Wada, Mikio

Watanabe, Takamasa

Watanabe, Yukiko

Weaver, Matthew

Yamada, Hiroyuki

Yamada, Nanako

Yamaguchi, Takashi

Yamaguchi, Yoshiko

Yamamoto, Shungo

Yamamoto, Shunji

Yamanashi, Hirotomo

Yamashita, Shun

Yano, Takahisa

Yodoshi, Toshifumi

Yokokawa, Daiki

Yokoya, Shoji

Yoshida, Eriko

Yoshida, Shuhei

Zukeran, Sota

